# Joining Forces: The Combined Application of Therapeutic Viruses and Nanomaterials in Cancer Therapy

**DOI:** 10.3390/molecules28227679

**Published:** 2023-11-20

**Authors:** Hongyu Li, Yunhuan Zhu, Xin Wang, Yilu Feng, Yuncheng Qian, Qiman Ma, Xinyuan Li, Yihan Chen, Keda Chen

**Affiliations:** 1 Shulan International Medical College, Zhejiang Shuren University, Hangzhou 310015, China; yunhuanzhu@outlook.com (Y.Z.); yilufeng1207@gmail.com (Y.F.); yunchengqian0831@gmail.com (Y.Q.); 202211001108@stu.zjsru.edu.cn (Q.M.); lxy2997388511@163.com (X.L.); yihanchen0411@gmail.com (Y.C.); 2 Ocean College, Beibu Gulf University, Qinzhou 535011, China; 3 Center of Infectious Disease Research, School of Life Science, Westlake University, Hangzhou 310024, China; wangxin17@westlake.edu.cn

**Keywords:** therapeutic viruses, nanomaterials, tumor treatment, drug carriers, combined application

## Abstract

Cancer, on a global scale, presents a monumental challenge to our healthcare systems, posing a significant threat to human health. Despite the considerable progress we have made in the diagnosis and treatment of cancer, realizing precision cancer therapy, reducing side effects, and enhancing efficacy remain daunting tasks. Fortunately, the emergence of therapeutic viruses and nanomaterials provides new possibilities for tackling these issues. Therapeutic viruses possess the ability to accurately locate and attack tumor cells, while nanomaterials serve as efficient drug carriers, delivering medication precisely to tumor tissues. The synergy of these two elements has led to a novel approach to cancer treatment—the combination of therapeutic viruses and nanomaterials. This advantageous combination has overcome the limitations associated with the side effects of oncolytic viruses and the insufficient tumoricidal capacity of nanomedicines, enabling the oncolytic viruses to more effectively breach the tumor’s immune barrier. It focuses on the lesion site and even allows for real-time monitoring of the distribution of therapeutic viruses and drug release, achieving a synergistic effect. This article comprehensively explores the application of therapeutic viruses and nanomaterials in tumor treatment, dissecting their working mechanisms, and integrating the latest scientific advancements to predict future development trends. This approach, which combines viral therapy with the application of nanomaterials, represents an innovative and more effective treatment strategy, offering new perspectives in the field of tumor therapy.

## 1. Introduction

Globally, the prevalence of tumor diseases continues to exert significant pressure on our healthcare systems, posing a serious threat to human life and health. As one of the main contributors to the global disease burden, cancer significantly impacts patients’ quality of life and survival rates. According to the World Health Organization statistics from 2020, there are approximately 19 million new cancer cases and about 10 million cancer-related deaths annually worldwide. Despite enormous progress in cancer diagnosis and treatment over the past few decades—such as more precise diagnostic techniques and more personalized treatment methods—many difficulties remain. In particular, achieving precision in cancer treatment, minimizing side effects, and enhancing therapeutic efficacy are key issues that continue to capture the attention of researchers and clinicians.

In cancer treatment, novel therapeutic approaches such as CRISPR/Cas9, Zinc Finger Nucleases (ZFNs), Cre/Lox gene editing, and immunotherapies like CAR-T and PD-1/PD-L1 inhibitors have emerged, yet they come with their challenges. Gene therapies may present safety and delivery concerns, as well as ethical and legal quandaries. Immunotherapies, while promising, have limitations; they are not suitable for all patients, may lead to resistance and immune-related adverse effects, and are often associated with high costs.

In recent years, the emergence of oncolytic viruses (OVs) and nanomaterials has breathed new life into the field of cancer treatment, offering renewed optimism in our battle against cancer. OVs, due to their natural tumor-targeting properties, possess the ability to accurately locate and attack cancer cells. Nanomaterials, within the domain of medical biotechnology, exhibit attributes such as biocompatibility, surface customization, and versatility. They serve as drug carriers, delivering therapeutic drugs directly and effectively to tumor tissues and reducing damage to healthy tissues. In the process of treating cancer, nanomaterials augment their biological distribution and boost their ability to penetrate targeted areas. Therefore, the combination of these two strategies has resulted in a new form of cancer treatment, namely ‘virus-nano complex therapy’. While OVs serve as a type of biological therapy, their stability and precision within the body often demand enhancement. Nanomaterials offer the potential to envelop and safeguard these OVs, ensuring their targeted delivery to tumor cells and controlled release. The synergistic use of nanomaterials with OVs significantly improves drug stability, bioavailability, and specificity.

In this article, we will delve into the application of therapeutic viruses and nanomaterials in cancer treatment, dissect their mechanisms of action in detail, and summarize the latest advancements. We aspire to offer substantial insights into this area.

## 2. The Formation of Tumors and Novel Treatment Strategies

### 2.1. The Occurrence of Tumors

Tumors are neoplasms formed by the abnormal proliferation of local tissue cells in the body, due to the loss of normal growth regulation at the genetic level under various carcinogenic factors [1]. Tumors are classified as either benign or malignant. Benign tumor cells proliferate at a slower pace, expand in a structured fashion, and typically neither invade adjacent tissues nor spread to distant body sites [2]. In contrast, malignant tumors—commonly referred to as cancer—are tumors with invasive characteristics. The growth and division of these tumor cells are out of control and are highly likely to invade surrounding tissues via the blood or lymphatic system to form new tumor foci [3].

As a complex disease, the treatment methods for tumors are diverse, including surgery, radiotherapy, chemotherapy, and targeted therapy. Compared with traditional treatment methods such as surgical removal, radiotherapy, and chemotherapy, the existing novel immunotherapies and gene therapies bring more possibilities to improve the quality of life and treatment outcomes for cancer patients. Therefore, strengthening cancer prevention, early screening and the rational use of various treatment schemes to improve treatment effectiveness is an important issue urgently to be addressed in the field of global public health.

### 2.2. Novel Therapies for Tumors

#### 2.2.1. Gene Therapy

CRISPR/Cas9, known for its precision in gene editing, has garnered significant attention in cancer therapy research. Michels and collaborators have utilized CRISPR/Cas9 for the pooled genetic screening of human colon organoids, revealing its high-throughput potential in pinpointing therapeutic targets for colorectal cancer [4]. It has been further exploited by Zhang et al. to engineer CAR-T-cells with viral transduction, marking a leap forward in CAR-T therapy innovation [5]. Complementing this, Rosenblum and associates have integrated CRISPR/Cas9 with cutting-edge nanoparticles, thus boosting the efficiency and precision of gene editing, which paves the way for advancements in tumor treatments and supports the broader application of gene therapy [6]. Transcription Activator-Like Effector Nucleases (TALENs), sharing CRISPR/Cas9’s precision but distinct in design, have been effectively employed by Jo et al. to modify endogenous TRAC and B2M genes. Their work resulted in the production of T-cells devoid of TCRαβ and HLA-ABC. These cells showed improved survival under immune surveillance, enhanced anti-tumor activity, and increased resistance to NK cell-mediated cytotoxicity [7]. Advancing the field, Das et al. crafted UCAR T-cells that target the CAF marker FAP, demonstrating capabilities in immune evasion and allogeneic reactivity [8]. The precision of TALENs has been further validated by their use in assessing the sensitivity of primary mouse tumor cells to doxorubicin, thereby reinforcing the value of TALENs in probing the chemotherapeutic response of tumors [9]. Moreover, ZFN and the Cre-Lox system have established themselves as formidable tools in genome editing. The application of ZFN has facilitated gene activation and repair, notably in restoring the function of p53 [10] and in formulating strategies to combat cervical cancer [11]. Although Vannocci et al. successfully demonstrated gene correction with ZFNs in cellular models [12], they acknowledged the need for advancements in primary stem cell applications. Employing ZFNs, Tang et al. achieved integration of CAR cDNA targeting EpCAM into iPSCs, leading to the generation of iNK cells adept at tumor targeting [13]. The Cre-Lox system’s precision has been substantiated through its application in numerous cancer studies, including the activation or deletion of genes in colon adenomas [14] and colorectal cancer [15] and in exploring the role of NSUN2 in the progression of pancreatic cancer [16]. This highlights its critical role in precise genomic regulation. Utilizing this system, Loesch et al. performed a targeted knockout of exon 3 of the CTNNB1 gene in hepatocytes, establishing a model that closely mirrors human liver cancer [17]. RNA interference (RNAi) technology, due to its high specificity, has shown potential for personalized therapy in suppressing oncogenes [18]. RNAi therapy based on plasmonic nanoparticles has been developed for precise cancer diagnostics and photothermal synergistic treatment [19], while redox-responsive nanoplatforms for specific siRNA have pioneered a new pancreatic cancer therapy by regulating tumor metabolism and immune function [20].

In addition to these gene-editing strategies, specific viral vectors such as lentiviruses and adenoviruses have played a pivotal role in gene therapy. They are capable of precisely delivering therapeutic genes into cancer cells, facilitating their intracellular expression, thereby inhibiting the progression of cancer [21]. However, gene-editing techniques such as these, while promising, carry risks such as off-target mutations and high costs, which may provoke immune responses and legal and ethical issues, and their effectiveness can be limited in certain tumors [22,23].

#### 2.2.2. Immunotherapy

Over the past several years, immunotherapy has achieved remarkable clinical results in treating various types of cancers. The approach revolves around leveraging the patient’s immune system to identify and eradicate cancerous cells, showing particularly impressive efficacy in the treatment of late-stage cancers [24].

One such strategy involves the use of immune checkpoint inhibitors [25,26]. These therapies function by weakening the tumor cells’ suppression of the immune system, thereby activating immune cells to more effectively attack the tumor cells.

**Figure 1:**
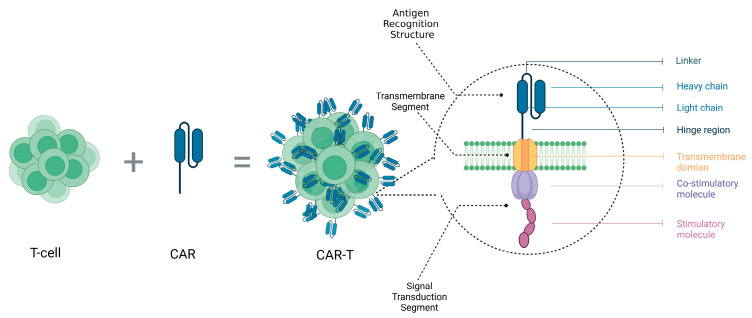
Schematic Diagram of CAR-T Structure. CAR typically consists of the following key components. ➀ Antigen Recognition Structure: this part is located outside the cell and is capable of recognizing and binding to specific antigens on tumor cells. It usually originates from the variable region of antibodies and has high antigen specificity. ➁ Transmembrane Segment: this part is a protein fragment that is responsible for anchoring the CAR to the T-cell membrane. ➂ Signal Transmission Segment: this part is located inside the cell and can trigger activation signals in T-cells. The original CAR had only one signal transmission domain, known as the CD3ζ chain, which came from the T-cell receptor complex. In the subsequently developed second and third-generation CARs, one or more co-stimulatory domains, such as CD28 or 4-1BB, were added. These co-stimulatory domains can enhance T-cell activity and longevity.

Another therapy showing significant clinical outcomes is CAR-T-cell therapy, whose structure is depicted in Figure 1. CAR-T involves genetically engineering T-cells to express tumor-specific chimeric antigen receptors and infusing these modified T-cells into the patient, thereby enabling activated T-cells to kill tumor cells. Oncolytic virotherapy is another major type of immunotherapy, which infects and kills tumor cells using genetically engineered viruses. Simultaneously, this therapy can also trigger an immune response, further eliminating residual tumor cells.

Currently, scientists are continuously optimizing existing immunotherapies and exploring new treatment strategies. This includes enhancing immunotherapy by regulating immune suppressive or promoting factors in the tumor microenvironment [27]. Furthermore, the integration of biomaterials with immunotherapies represents a novel research avenue. These biomaterials act as carriers for immune cells or vaccines, enhancing the stability and improving the delivery efficiency of immunotherapeutic treatments.

## 3. Therapeutic Viruses

Since the 1950s, the view of viruses solely as harmful has evolved as researchers began exploring their use in immunotherapy. Initially used as tools in disease research, therapeutic viruses soon showed promise for medical treatments. It witnessed the emergence of viral vectors for gene transfer in the 1970s, highlighting their potential to treat diseases. By the 1990s, these vectors had advanced to clinical trials, overcoming initial technical challenges. The early 2000s saw the development of OVs targeting cancer cells, with recent progress highlighting herpes virus efficacy in colorectal cancer therapy. Currently, various therapeutic viruses are deployed in oncology, selectively targeting and lysing tumor cells with minimal impact on normal tissues. Several have entered clinical trials or have been approved for cancer treatment, as summarized in Table 1.

### 3.1. Oncolytic Viruses

**Figure 2:**
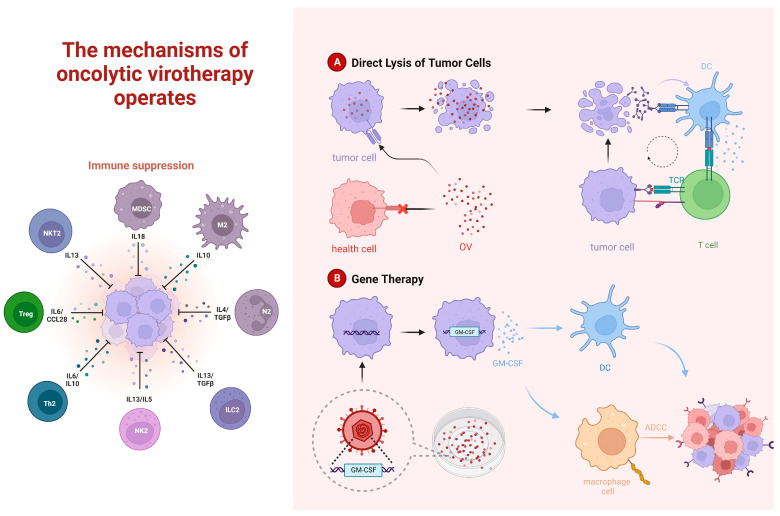
Tumors are capable of creating an immunosuppressive environment, effectively evading the surveillance of the immune system, and thereby facilitating growth, dissemination, and metastasis. For instance, they could alter their surface proteins to evade recognition by the immune system or secrete immunosuppressive molecules such as cytokines (like TGF-β and IL-10) to directly inhibit the activity of immune cells. Tumor cells can even recruit and activate certain immunosuppressive cells, such as regulatory T-cells (Tregs) and myeloid-derived suppressor cells (MDSCs). These cells can suppress immune cells, preventing them from attacking the tumor. Presently, OVs primarily employ two strategies to break through this immunosuppressive environment. (**A**), Direct cell lysis. OVs selectively infect tumor cells, leading to their lysis and the release of more antigens, which further intensifies the immune response and specifically induces tumor cell apoptosis. (**B**), Gene therapy. For example, OVs carrying the GM-CSF gene infect tumor cells in large numbers, causing the tumor cells to express GM-CSF. This recruits immune cells such as dendritic cells and macrophages, induces immune infiltration, and destroys pathogens or tumor cells that have been marked by antibodies.

The concept of OVs could be traced back to the 1950s. At that time, scientists discovered a virus named “Adenovirus 12” that could cause tumor dissolution in mice. Subsequently, researchers discovered a series of viruses with oncolytic properties and attempted to harness them for clinical treatments. OVs are a class of viruses that are capable of infecting and specifically destroying cancer cells, with relatively minimal impact on normal cells [35]. The basic principle of OV therapy in cancer treatment involves three primary mechanisms. Firstly, after OVs infect cancer cells, they reproduce and release new viral particles, leading to cancer cell death and the activation of the anti-tumor immune response [36,37]. The virus infection induces cancer cells to express antigens, making it easier for immune cells like T-cells and NK cells to recognize and attack cancer cells [38]. Secondly, some OVs stimulate the production of anti-tumor cell factors, enhancing the body’s immune clearance of cancer cells [39]. Additionally, OVs interfere with the growth and division signals of cancer cells, thereby inhibiting tumor development [40]. The related mechanisms are presented in Figure 2.

OV therapy, an emerging method in cancer treatment, boasts numerous advantages. It targets cancer cells selectively, enhances the immune system, employs various therapeutic mechanisms, offers tailored treatment, and is suitable for many cancer types. Moreover, it can be integrated with other therapies to extend its benefits, reduce adverse effects, and combat chemoresistance [41]. A notable example is its combination with cytokine-induced killer (CIK) cells, a novel type of immunotherapy, which, in conjunction with Oncolytic Adenovirus (OAds), has shown promising effects in cancer treatment [42].

However, OV therapy is not without limitations. The impact on non-cancerous cells is generally minimal, but it often leads to side effects like fever, fatigue, and muscle aches. There’s also the risk of the immune system neutralizing the therapeutic viruses, thereby diminishing the treatment’s effectiveness [43]. Patient responses to OV therapy also vary considerably, with some individuals unable to tolerate the treatment effectively [44]. Moreover, challenges such as uneven distribution within the body, significant toxic side effects, and lack of lasting presence highlight the urgent need for efficient delivery systems. The swift advancement in combining OVs with various carriers and materials is opening up new avenues to address these challenges in OV therapy.

#### 3.1.1. Oncolytic Viruses Combined Radiochemotherapy

The combined application of OVs and radiochemotherapy has become a hot topic in the field of cancer treatment. Radiochemotherapy, a traditional cancer treatment approach, effectively eradicates tumor cells, but it may also harm healthy cells. In contrast, OVs target and obliterate cancer cells with precision, minimizing damage to normal cells.

When OVs and radiochemotherapy are used in combination, they could mutually enhance each other’s therapeutic effects. Firstly, radiochemotherapy may reduce the immune suppression effect of tumor cells, increasing the infection rate of OVs in tumor cells [45]. Furthermore, the infiltration of OVs induces apoptosis and necrosis in tumor cells, which may facilitate the deeper penetration of radiochemotherapeutic agents into the tumor mass, thus potentiating their effectiveness [46]. Additionally, OVs enhance anti-tumor immune responses. When combined with radiochemotherapy, they aid in overcoming tumor resistance to treatment. OVs have gained widespread use in combating various cancers, presenting significant benefits compared to conventional methods. They also complement radiotherapy and chemotherapy, amplifying the therapeutic impact. A genetically modified Vaccinia virus, GLV-1h153, has been demonstrated to have good therapeutic effects and preventive ability against metastasis, offering the potential for a successful treatment approach to triple-negative breast cancer [47].

The research conducted by Fan and colleagues revealed that the synergistic application of Oncolytic Herpes Simplex Virus (HSV) and Temozolomide (TMZ) resulted in diminished viability of breast cancer cells, arrest of the cell cycle, induced apoptosis of tumor cells, and heightened DNA damage response in vitro [48]. In a Phase 1 dose escalation trial, patients with primary high-grade gliomas had a median overall survival of 18.4 months after surgical removal of the tumor and the combined use of engineered OAds with Temozolomide and radiotherapy. The results showed the feasibility and safety of the combined therapy [49].

Nevertheless, the combined application of OVs and radiochemotherapy also faces some challenges in practical use, such as virus safety and virus clearance issues. Therefore, scientists are actively studying to improve this combined treatment strategy, in hopes of bringing better treatment effects for patients in the future.

#### 3.1.2. Oncolytic Viruses Combined with Immune Checkpoint Therapy

The recent surge in oncological research has spotlighted the combined use of OVs and immune checkpoint inhibitors in cancer treatment. Immune checkpoint inhibitors, including Programmed Cell Death Protein 1 (PD-1)/Programmed Death-Ligand 1 (PD-L1) and Cytotoxic T Lymphocyte-Associated Antigen 4 (CTLA-4) inhibitors, invigorate the immune response against tumors by disrupting the suppressive signals exchanged between tumor and immune cells. Their effectiveness and precision receive a significant boost when combined with OVs.

Immune checkpoints are mechanisms that regulate the intensity and duration of immune responses through inhibitory pathways. This mechanism involves cytotoxic T lymphocyte-associated antigen 4, programmed cell death PD-1, and PD-L1, which negatively regulate activated T-cells and NK cells. It helps maintain immune balance and protects the body from autoimmune damage [50,51]. However, tumor cells also exploit this inhibitory pathway to evade the attack of the immune system. In response, scientists have developed inhibitors against immune checkpoints, such as Ipilimumab, Nivolumab, and Pembrolizumab. These inhibitors can block the suppressive effects of tumor cells on immune cells, thereby enhancing anti-tumor immunity [52,53].

Focusing on the PD-1/PD-L1 pathway, inhibitors targeting this route can prolong T-cell activity and rejuvenate their ability to combat cancer cells [54]. The strength of integrating OVs with immune checkpoint inhibitors lies in their synergistic effects, amplifying treatment efficacy. By infecting tumor cells, OVs release antigens and trigger immune cell activation, thereby augmenting the impact of immune checkpoint inhibitors [43].

Simultaneously, immune checkpoint inhibitors diminish the suppression of immune cells by tumor cells, then enhance the anti-tumor immune response elicited by OVs’ infection [55]. This combination treatment strategy has shown potential efficacy in clinical trials, achieving better treatment results for certain types of cancer patients.

The efficacy of anti-PD-1/PD-L1 treatments, while promising, remains suboptimal, hindered by various factors such as PD-L1 expression levels in the tumor microenvironment, lymphocyte infiltration, T-cell receptor clonality, tumor neoantigens, and peripheral blood markers.

Currently, single immune checkpoint inhibitors are not effective for all patients and sometimes may not provide satisfactory results. This may be related to the expression of immune checkpoint molecules on the surface of tumor cells and the degree of immune cell infiltration. Immune checkpoint inhibitors are more effective for certain types of tumors (such as melanoma, lung cancer, and renal cell carcinoma) but may be less effective for other types. Various factors in the tumor microenvironment, such as cells, cytokines, and growth factors, can affect the efficacy of immune checkpoint inhibitors. For instance, suppressive cells in some tumor microenvironments (such as regulatory T-cells, myeloid-derived suppressor cells, etc.) may inhibit the immune response, reducing drug efficacy [56]. Immune checkpoint inhibitors may cause immune-related adverse effects, such as pneumonia, hepatitis, colitis, dermatitis, etc. [57]. These side effects may prevent patients from continuing to use the drug, thus affecting efficacy. Therefore, the potential anti-tumor effects of single immune checkpoint inhibitor therapy are hard to achieve, and the combination with OVs may yield better results.

**Figure 3:**
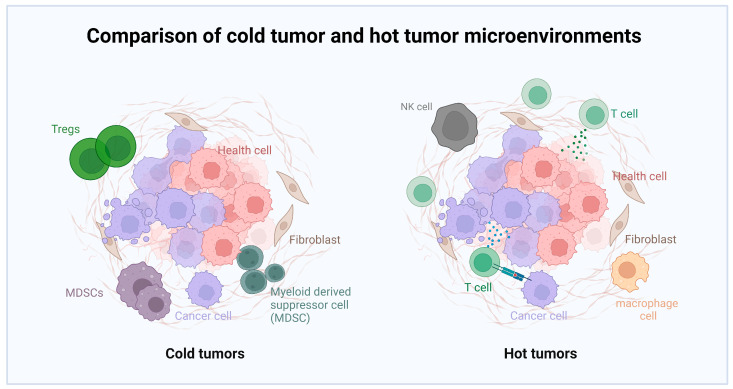
The microenvironments of ‘cold’ and ‘hot’ tumors greatly differ. Cold tumors usually lack effective T-cell infiltration and might lack tumor-specific antigens, making it challenging for the immune system to recognize and attack these tumors. Moreover, the microenvironment of cold tumors may be rich in immunosuppressive cells, such as regulatory T-cells (Tregs), M2 macrophages, and myeloid-derived suppressor cells (MDSCs). These cells further inhibit immune responses, enabling the tumor to more effectively evade the immune system’s attack. Besides, hot tumors typically exhibit substantial immune cell infiltration, particularly tumor-specific T-cells. These tumors usually possess many tumor mutation antigens, making them more easily recognized and attacked by the immune system. Besides T-cells, other immune cell infiltrations, such as B cells, NK cells, dendritic cells, and M1 macrophages, may also be present in hot tumors.

Studies have shown that OV therapy in combination with anti-PD-1/PD-L1 leads to a synergistic effect. OVs possess the capability to transform the tumor immune microenvironment, converting ‘cold’ tumors to ‘hot’, thus rendering previously immune-suppressive tumors sensitive to immune checkpoint inhibitor therapy. This combination therapy is biologically logical because OVs can reverse immune suppressive factors in tumor cells, induce inflammatory immune infiltration, and create new tumor antigen epitopes caused by genetic mutations, leading to specific tumor T-cell responses, as shown in Figure 3. For example, studies have found that Zika virus (ZIKV) could induce strong pro-inflammatory responses and increase CD4+ and CD8+ T-cell tumor infiltration and activation in a mouse model of Glioblastoma Multiforme (GBM) [58].

On the other hand, virus-mediated oncolysis induces high-level expression of the antigen-presenting cell PD-L1 in tumor-infiltrating lymphocytes, making the tumor more receptive to systemic treatment with immune-modulatory antibodies. Research has shown that Chimeric Vaccinia Virus CF33 expressing anti-PD-L1 antibody can infect pancreatic ductal adenocarcinoma cells, producing anti-PD-L1 antibodies, effectively blocking the action of PD-L1 on the surface of tumor cells, leading to the enhanced anti-tumor immune killing [59].

Extensive research indicates that integrating OVs with anti-PD1/PD-L1 therapy prolongs survival in mice with tumors and amplifies the anti-tumor efficacy. Regarding drug toxicity, the combination of OVT-VEC and Ipilimumab in clinical trials does not increase the toxicity of monotherapy [60].

In summary, the combination of OVs with anti-PD-1/PD-L1 therapy has the potential to enhance anti-tumor effects. However, this combination therapy still faces many challenges, such as individual differences in efficacy, drug toxicity, and potential anti-viral immune responses. Future research needs to further explore the best application strategies for this combination therapy and patient selection criteria to improve treatment outcomes and reduce side effects.

Although combining OVs with immune checkpoint inhibitors holds great promise for cancer treatment, several challenges remain. These include selecting the optimal viral vector, determining the appropriate dosage and timing for virus release, and understanding the interactions between the virus and immune checkpoint inhibitors. With ongoing research, it is hopeful that these challenges can be overcome in the future, providing cancer patients with more effective and safer combination treatment strategies.

#### 3.1.3. Oncolytic Viruses Combined with Adoptive Cell Therapy

Combining OVs with Adoptive Cell Therapy (ACT) is an innovative approach to cancer treatment. This strategy aims to enhance the efficacy of cancer treatments by utilizing OVs to disrupt tumor cells, thereby releasing tumor antigens, reversing tumor immune suppression, and stimulating anti-tumor immune responses [61]. ACT provides patients with a large number of tumor-specific immune cells, aiding in the eradication of residual cancer cells.

Moreover, OVs infecting tumors lead to alterations in the tumor microenvironment, thereby enhancing immune cell infiltration and activation. In addition, OVs stimulate natural T-cell receptors, which improves CAR-T-cell targeting of the tumor [62]. By combining OVs with ACT, a synergistic effect is achieved, delivering a dual assault against cancer cells.

In solid tumors, due to the lack of infiltration and persistence of CAR-T-cells in tumor tissues, the therapeutic effect is greatly compromised. Huang developed an OAd carrying Interleukin-7 and CAR-T targeting B7H3. The study evaluated the in vitro and in vivo efficacy of OAd-IL7 and B7H3-CAR-T used separately or in combination with glioblastoma. The improved anti-tumor effect of CAR-T was assessed based on T-cell proliferation, survival, persistence, exhaustion, and tumor regression. Results showed that mice receiving both OAd-IL7 and B7H3-CAR-T treatment exhibited prolonged survival and reduced tumor burden [63]. Other research addressed the limitation of the lack of tumor-specific and uniformly expressed tumor antigens in solid tumors. This was completed by designing an OV expressing a non-signaling, deficient CD19 (CD19t) protein, enabling tumor-selective transmission and targeting by CD19/CAR-T-cells. In the study, the OV19t infected tumor cells, generating new CD19 on the cell surface before virus-mediated tumor lysis. When co-cultured with CD19/CAR-T-cells, they secreted cytokines and showed strong cytotoxic activity against the infected tumor [64].

A study examining the infection and replication of LOAd703 OAd in B-cell lymphoma cell lines demonstrated that LOAd703 could infect these cells and induce the enhancement of immunogenic profile, upregulating the expression of costimulatory molecules, MHC molecules, etc. The results indicated improved CAR-T-cell functions in cytokine release and lymphoma cell killing. Moreover, LOAd703-infected lymphoma cells also upregulated the secretion of some chemokines, which could enhance the migration of CAR-T-cells [65].

The aforementioned studies indicate that the combination of OVs with ACT holds potential efficacy in various types of cancers. However, it is important to note that most of these studies are confined to laboratory and animal models. Although some studies have advanced to clinical trials, further validation of their safety and effectiveness is required. Moreover, researchers need to optimize treatment plans and dosages for different cancer types and individual differences to achieve better therapeutic outcomes.

#### 3.1.4. Oncolytic Viruses Combined with Pretreatment of Nanomaterials

To overcome the obstacles encountered in clinical applications, such as the stability of viruses, their specific infectivity, and evasion of the host immune system’s rapid clearance, researchers have developed various nanotechnology-based pre-treatment methods.

Nanocarriers such as liposomes and polymeric nanoparticles encapsulate OVs, which not only enhance the virus’s stability within the organism but also promote its diffusion and release [66]. Liposomes, such as CCL2 and polyethylene glycol (PEG) are commonly used nanocarriers for wrapping OVs [67,68,69], including the use of liposome-encapsulated telomerase-specific OAd (TelomeScan), carrying plasmid DNA expressing green fluorescent protein (GFP) (Lipo-pTS) as a potential method for systemic delivery of OVs [70]. Recently, an OAd vector encapsulated in oncolytic nanospheres has been developed through a multifunctional protein surface precipitation (PSP) technique, which supports the nanospheres’ spontaneous targeting of lung cancer cell lines (A549) [71]. The nanospheres maintain their structural stability through surface charge and structural nodules and exhibit unique tetrahedral or hexagonal morphologies. Employing the PSP technique boosts viral transfection efficiency and may simultaneously diminish the T-cell immune response to the PSP technique during in vivo transport. This reduction in immunogenicity helps to preserve the bioactivity of OAds following their release [72,73]. Additionally, Naseer et al. are developing a novel green nanomedicine using thiolated chitosan coated with hyaluronic acid, specifically designed for targeted delivery of an oncolytic measles virus (OMV) vaccine strain to treat prostate cancer [74], Notably, extracellular vesicles are also being studied as a drug delivery tool for cancer therapy [75].

To enhance the selectivity and infectivity of OVs, a series of surface-targeting ligand modification techniques have emerged. For instance, PEG modification (PEGylation) could reduce the likelihood of the virus being recognized by the host immune system, thereby prolonging its survival time in the bloodstream. Further, hexon modification has been utilized to augment the specificity and activity of OAds towards pancreatic cancer cells and stromal cells [76]. Moreover, research by Jung et al. has demonstrated that encapsulating OAds expressing tumor necrosis factor-related apoptosis-inducing ligand (TRAIL) within gelatin hydrogels (OAd-TRAIL/gel) effectively enhances and prolongs the virus’s anti-tumor action [77].In another study, the combination of chitosan-PEG-folic acid (FA) nanocomplexes with OAds significantly improved the therapeutic effect and safety against folic acid receptor (FR)-positive cancers. This not only extended the virus’s circulation time in the blood but also reduced its accumulation in the liver, enhancing the possibility of systemic administration [78]. Additionally, scientists have noted that ursolic acid nanoparticles (UA-NP) prepared via nanoemulsion techniques can improve solubility and bioavailability and exhibit a synergistic effect in enhancing the apoptotic cytotoxicity of oncolytic measles virus (MV) against breast cancer cells, indicating potential for breast cancer therapy and clinical advantages of ursolic acid [79]. Furthermore, the insertion of Ad.SPDD-HCCS1, expressing the cassette AFP-HCCS1-WPRE-SV40, has improved the safety and efficacy of oncolytic-mediated liver cancer gene therapy [80]. Enzymatic modifications, such as histone deacetylase inhibitors (HDACis), have also been proven to enhance virus replication and the efficacy of oncolytic HSV-1 therapy. For example, treatment with trichostatin A (TSA) for oral squamous cell carcinoma (SCC) can activate nuclear factor-kappa B (NF-kB), thereby enhancing the replication of gamma(1) 34.5-deficient oncolytic HSV-1 (oHSV) [81]. iRGD tumor-penetrating peptide-modified OAds have shown enhanced tumor transduction, intratumoral spread, and anti-tumor effects [82] Lastly, oncolytic vaccinia virus (OVV) coated with erythrocyte-derived membranes (EDM) is expected not only to circulate longer in vivo, similar to intravenously injected erythrocytes, but also to respond to environmental pH changes, due to their unique properties [83].

Conversely, the fusion of OVs with magnetic nanoparticles is a burgeoning frontier in cancer therapy. These nanoparticles can induce hyperthermia or generate reactive oxygen species within the tumor vicinity, both directly obliterating tumor cells and facilitating the OVs’ infection and proliferation. By adopting this method, the local therapeutic effect of the OVs is intensified, while also reducing the side effects typically seen with systemic treatments. Choi et al. have developed a technique involving PEG-coated magnetic iron oxide nanoparticles (MIONs) for encapsulating OAds [84]. The employment of an external magnetic field (EMF) to steer these nanoparticles markedly enhances the precision and infection rates of OAds, addressing the challenges of non-specific targeting and tumors’ low viral receptor expression [85,86]. Meanwhile, Wu and colleagues have crafted a novel “cell robot” using 293T cells. These cellular automatons, laden with OVs and adorned with targeting peptides and magnetic asymmetric Fe_3_O_4_ nanoparticles, accomplish dynamically targeted delivery under magnetic guidance. This innovation enables the precise identification and eradication of bladder cancer cells [87].

Through these nanoparticle-based pre-treatment methods, the efficacy of oncolytic virus therapy has been significantly enhanced. However, each approach has its specific indications and limitations. Currently, these strategies are in continuous development and optimization stages and require further laboratory research and clinical trials to ensure their safety and effectiveness. The progress in this field indicates a broadening horizon for the application of OVs in cancer therapy.

### 3.2. Viral Vectors

Vector viruses are often the primary transport tool in gene therapy. Their main advantages lie in their ability to insert specific genes precisely, altering the behavior of cancer cells or enhancing the body’s immune response to cancer. This approach possesses high specificity and efficiency. However, the challenges with this strategy relate to safety and immune response. Some viruses might trigger intense immune responses or may induce undesirable side effects during the gene insertion process. The use of viruses as tools for treating cancer has been widely studied, with the design and successful application of viral vectors in cancer treatment being of milestone significance.

Gene therapy, as an emerging field in cancer treatment, primarily fights diseases by adjusting the genome of human cells. The application of viral vectors in gene therapy has been extensively studied. For instance, the hybrid viral vectors developed by Hajitou and colleagues [28], and the activation effects of replicating viral vectors on CD8(+) T-cells [30], have opened new possibilities for tumor immunotherapy. Viral vector-expressed TNF genes could induce apoptosis in tumor cells [31], further highlighting the application potential of viral vectors in gene therapy. At the same time, gene-virus combination therapy has demonstrated its advantages in enhancing the selective infection of the virus and boosting gene anti-tumor effects [32]. In clinical experiments, the application of systemic temozolomide-activated bacteriophage-targeted gene therapy in glioblastoma treatment has proven the effectiveness of bacteriophage vectors [33]. Moreover, the mediation of viral vectors can remodel the tumor stroma to achieve curative cancer treatment [88].

The research discussed has extensively examined the pivotal roles and immense potential of viral vectors in cancer therapy. These vectors have been utilized for targeting tumors with specific ligands, inducing anti-tumor immune responses, driving the expression of anti-tumor genes, and modifying the tumor microenvironment. Consequently, viral vectors have emerged as a crucial component in oncological treatments. However, ongoing research is essential to further refine these strategies, aiming for more effective and safer cancer therapies.

The primary benefit of drug-delivery viruses is their ability to directly convey therapeutic drugs or treatments to cancer cells. This precision targeting spares normal cells, thereby optimizing therapeutic outcomes and enhancing efficacy, while concurrently diminishing side effects. Nonetheless, these drug-delivery viruses may elicit immune responses in patients, potentially affecting their therapeutic effectiveness. Additionally, the selectivity of these viruses is not guaranteed; not all tumor cells are susceptible to effective infection and targeting. This limitation could restrict the wide-scale use of this approach in cancer therapy.

Ghosh and Banerjee developed an intelligent viral vector that specifically delivers hydrophobic drugs. This new type of viral vector can alter the drug distribution within the body, and improve the drug selectivity for tumor tissues, thereby enhancing the efficacy and safety of hydrophobic drugs [89].

In these treatment strategies, the virus is designed to carry drugs or other therapies, and then deliver them to cancer cells. These viruses could selectively infect cancer cells through specific biochemical mechanisms, thus delivering the drug directly to cancer cells, sparing normal cells. For example, a treatment known as cytotoxic drug-loaded adenovirus therapy has entered clinical trial stages. This approach uses adenovirus as a carrier to deliver chemotherapy drugs directly to tumor tissues. This method efficiently delivers the drug to tumor tissues, thereby reducing the drug’s distribution throughout the body, reducing side effects, and enhancing efficacy.

These therapies hold considerable potential, yet they face several challenges in clinical applications. In the future, we may need to develop new strategies to enhance the efficiency of these therapies, reduce side effects, and increase viral selectivity.

It’s worth mentioning that phages are a type of virus composed of a protein shell and nucleic acid (DNA or RNA). Proteins on the shell can bind to host cell receptors for targeting, allowing the phage to infect specific cells [90]. Therefore, when designing phage therapy for cancer, researchers can modify phages by screening for highly specific tumor-targeting peptides, allowing for precise targeting of tumor cells. This specificity helps reduce the impact on normal cells and improve therapeutic effects. Screening methods include phage display techniques and bioinformatics methods, which could identify peptides with high affinity binding to tumor cell surface receptors [91].

The life cycle of a phage typically involves five stages: adsorption, penetration, biosynthesis, assembly, and release. Understanding this lifecycle aids researchers in assessing the phage’s infection efficacy and destructibility toward tumor cells.

Although phages are not classified as OVs, their unique structure enables a novel anti-cancer therapy involving the combined use of phages and nanomaterials, which benefits from the strong affinity and high specificity of phage proteins. This specificity can increase drug concentration within tumor tissues, thereby enhancing therapeutic effects. For example, researchers have coupled phage proteins to nano-drugs via chemical bonds or physical adsorption, forming a composite structure. The phage proteins can guide the nano-drugs precisely to tumor cells, where the nano-drugs release anti-cancer drugs, achieving efficient therapeutic effects [92]. Overall, phage therapy has advantages in cancer treatment, such as strong targeting, low toxicity, and high customizability. However, it also points out some potential problems, like immune response, viral clearance, and drug delivery.

In summary, a range of experiments employing animal models have substantiated the efficacy and sustainability of using carrier viruses as a treatment modality for tumors. While these experiments open up avenues for further discussion, the innovative concepts they introduce are invaluable for further exploration in this field. They guide future research toward identifying critical factors that influence these experiments and towards expanding upon the experimental findings.

### 3.3. Immune Viruses and Virus-like Particles

Immunoviruses represent another approach to treating malignant tumors. Immunoviruses can carry relevant antigens, activate the immune system, and stimulate and induce cellular and humoral immunity to produce antibodies. The Human Papillomavirus (HPV) vaccine is an example of an immune virus, and it is currently widely used to prevent cervical cancer. Many other types of immunovirus vaccines have been shown to play a significant role in inhibiting tumor growth, such as the Vaccinia Virus vaccine.

However, the direct use of viruses as a treatment tool poses some safety concerns. This has led to the emergence of Virus-like particles (VLPs) as an attractive alternative strategy. The advantage of VLPs lies in their ability to mimic the form and structure of viruses, but without containing viral genetic material, they do not cause viral infections [93]. As a result, VLPs can be considered safer analogs of viruses.

In terms of VLPs preparation and immunogenicity, extensive research has shown that VLPs can be produced through genetic engineering or chemical synthesis among other methods, and they can effectively mimic real viruses, inducing a strong immune response. Detailed elaboration on these aspects has been made in a study by Nooraei and others, which found that, in addition to eliciting an immune response as nano vaccines, the produced VLPs can also serve as nanocarriers for drugs, further enhancing therapeutic effects [94].

Moreover, VLPs have played a significant role in vaccinology. As pointed out in an article by Mohsen and Bachmann, VLPs can be utilized not only as preventative vaccines but can also be developed into therapeutic vaccines for treating existing diseases [93]. Currently, a variety of VLP-based vaccines and drugs have entered clinical trial stages, all demonstrating good efficacy and high safety, further proving the significant value of VLPs in cancer treatment.

These studies propose the application of a carrier and gene-virus combined treatment strategy in cancer therapy from a preventative perspective, which could provide new directions for future cancer prevention and treatment.

## 4. Nanobiomaterials Delivery Systems

### 4.1. Definition and Advantages of Nanobiomaterials

Biomaterials are a class of materials that interact with biological entities and possess characteristics such as biocompatibility and biodegradability. They are widely used in fields like tissue engineering, biomedicine, and drug delivery. Bio-nanocarriers are a type of delivery system that encapsulates active components such as drugs, genes, and immunotherapies into nano-scale carriers using nanotechnology. These delivery systems are typically composed of biocompatible nanomaterials, such as liposomes, polymer nanoparticles, protein nanoparticles, etc. Given their size, typically between 1 and 100 nanometers, they offer better biological distribution, penetration capabilities, and targeting.

Bio-nanocarrier materials are made from biocompatible materials, thus they are less likely to trigger adverse reactions in the body, such as immune responses and inflammation [95]. In addition, these materials have biodegradable properties, allowing them to naturally degrade after performing their functions, avoiding complications from long-term accumulation. Bio-nanocarrier systems can achieve a higher drug payload, thereby improving drug efficacy [96]. Experiments have shown that using nanoparticles as carriers to deliver anti-tumor drugs to tumor vascular endothelial cells effectively blocks the formation and growth of tumor vessels [97]. With its one-of-a-kind size and the potential for precise molecular modifications, nanomedicine provides notable advantages for the focused delivery of therapy at the site of lesions [98]. Through design and optimization, bio-nanocarrier systems can even realize controlled drug release, thus releasing drugs at the appropriate time and place to enhance therapeutic effectiveness. Nano-delivery can enhance the stability and solubility of drugs, increase drug distribution and penetration in the body [99], improve drug bioavailability, prolong drug half-life, and reduce drug toxicity and side effects. In the treatment of metastatic breast cancer, the nano-delivery system can precisely deliver drugs to tumor cells and improve therapeutic effects by enhancing drug pharmacokinetics, increasing targeting, and improving drug stability [100].

Bio-nanocarrier materials hold significant advantages in drug delivery and gene therapy fields. These advantages are primarily manifested in biocompatibility, biodegradability, high drug loading, controlled release, improved drug bioavailability, reduced drug toxicity and side effects, and enhanced targeting, among other aspects. Some commonly used materials are shown in Table 2.

### 4.2. Nanobiomaterials

Liposomes, resembling cell membranes with their phospholipid vesicle structure, effectively encapsulate and deliver both hydrophilic and hydrophobic drugs directly into cells through endocytosis, targeting specific intracellular sites [106,107,108,109]. They possess the ability to cross formidable biological barriers, including the blood-brain barrier, enabling precise treatments [110], and can be engineered to bind selectively to cell receptors, thus improving targeted drug delivery [111]. This technology is exemplified in mRNA-LNP therapies that utilize synthetic mRNA for protein synthesis and immune activation [112], and in advanced liposomal drugs like Doxil^®^/Caelyx^®^ (Janssen Products, LP, New Brunswick, NJ, USA or Schering-Plough Corporation, Kenilworth, NJ, USA), DaunoXome^®^ (Galena Biopharma, San Ramon, CA, USA), and Onivyde^®^ (Ipsen Biopharmaceuticals, Inc., Basking Ridge, NJ, USA), which are landmarks in cancer therapy due to their specificity and enhanced efficacy.

Mesenchymal Stem Cells (MSCs), known for their self-renewal and differentiation abilities in tissues like bone marrow and umbilical cord, play crucial roles in tissue repair and immunoregulation and are increasingly studied for treating cardiovascular and neurological disorders [113]. Research has shed light on MSC-derived extracellular vesicles (MSC-EVs), ranging from 30 to 1000 nanometers and packed with proteins, lipids, and nucleic acids, which facilitate intercellular communication and content delivery through surface protein interactions and signal transduction [114,115,116,117,118]. With low immunogenicity and the ability to cross barriers like the blood-brain barrier, MSC-EVs present a safer, more stable alternative to MSCs for delivering therapeutic agents, including anticancer drugs. Their ease of preservation and straightforward clinical application hint at promising therapeutic outcomes for various diseases [119,120].

Advancements in nanoparticle research have led to innovative nanomedical technologies surpassing conventional materials, significantly advancing the efficiency of nanoparticle-mediated drug delivery and tumor diagnostic and treatment modalities, including imaging and photothermal therapy (PTT) [121,122]. Research shows that negatively charged nanoparticles excel in tumor penetration due to their higher diffusion coefficients [123]. Modifying nanoparticle characteristics like potential and size, exemplified by red-light-induced size reduction techniques, has been shown to enhance tumor infiltration and intracellular uptake [124] Hyaluronic acid-based nanoparticles have proven particularly potent in delivering miRNA to the Tumor Microenvironment (TME), offering targeted cancer therapy solutions [125].

Emerging nanoscale imaging methods are outperforming traditional imaging technologies (X-ray, CT, MRI, ultrasound, PET) by leveraging enhanced nanocarriers to improve specificity, sensitivity, and safety. Notably, folate-engineered nanocarriers for synchronous imaging and therapy [126]. porphyrin-based nanoparticles for fluorescence imaging [127], and silica-gold nanoshells for precise therapeutic monitoring [128] are enhancing cancer detection and treatment strategies. Moreover, gold nanorod-based nanohybrids have escalated tumor imaging precision through their multimodal capabilities [129].

In PTT, Zhu et al.’s development of blue-light responsive *E. coli*, combined with nanoparticles [130], and Gao et al.’s construction of cell membrane-anchored nano photosensitizers for tumor-specific calcium overload induction [131] exemplifies the integration of nanotechnology with optogenetics and immunotherapy. The application of Keratin-wrapped gold nanoparticles within 3D bioprinting technologies facilitates targeted PTT [132]. while FA-AuNPs and functionalized nanoparticles like PDA-OPN and GNR-SFE enhance the efficacy of cancer cell ablation [133,134].

Finally, nanotechnology’s role in biomarker detection has been amplified, with novel photothermal sensors suitable for integration with smartphones for enhanced clinical diagnostics [135]. Practical applications include graphene oxide nanoparticles in MRI for PTT impact monitoring [136], and DNA nanorobots for intelligent therapeutic delivery, opening new avenues in cancer treatment [137]. These innovations advance cancer treatment technologies and simultaneously pave new pathways for cancer detection.

### 4.3. Drawbacks and Limitations

In contrast, there are some disadvantages to this technology. The production cost of nano-materials is high, limiting large-scale production and application. Regarding usage, the instability of nano-materials in the body may lead to degradation and inefficiency. Some nano-delivery systems may cause adverse reactions in biological tissues, leading to overactive immune responses or cytotoxicity [138]. Furthermore, some nano-delivery systems have inadequate drug targeting, potentially leading to drug accumulation in non-target tissues, and causing severe side effects.

## 5. Combination of Therapeutic Viruses and Nanobiomaterial Delivery Systems

### 5.1. Combination of OVs and Nanobiomaterials

#### 5.1.1. Mechanism Principles and Advantages

The integration of nano-delivery technology with OVs significantly enhances their precision in targeting. Utilizing nanoparticles like liposomes and extracellular vesicles for drug encapsulation and delivery fine-tunes this approach [139]. These nanoparticles act as carriers, ferrying OVs straight into tumor cells, thus improving the targeting accuracy and biodistribution. OVs then proceed to decimate tumor cells by invasion and release of replicative viral particles [140]. This combined strategy not only bolsters the oncolytic efficacy but also curtails adverse reactions, thereby advancing therapeutic outcomes.

The combination of the two endows the antitumor drugs with the advantages of both OVs and nano delivery technology, allowing the antitumor drugs to maintain high stability and bioavailability while killing tumor cells, stimulating the host immune system, reducing tumor cell drug resistance, and improving the effectiveness of the treatment. Through surface modifications, including the use of tumor-specific antibodies, ligands, peptides, sugars, etc., the targeting and specificity of OVs are enhanced, enabling the nanoparticle carriers to deliver drugs more accurately to tumor cells, while greatly reducing the impact on healthy cells [141,142].

#### 5.1.2. Research Progress

Liposomes are tiny vesicles composed of phospholipids cholesterol, etc. The unique vesicle structure of liposomes allows them to be widely used in drug delivery, gene therapy, and vaccine development. Liposomes can interact easily with cells through their cell-membrane-like structure, achieving efficient delivery of drugs, genes, and vaccines [109,143]. They possess functions such as biocompatibility, drug protection, controllable drug release, and targeted delivery.

Thanks to the enhanced permeability and retention effect of tumors: for tumor cells, due to their abnormal vascular system, high vascular density, and larger gaps between cells, certain molecules or nanoparticles have higher permeability and retention time in the tumor region [144], allowing nanoparticles and drugs to pass through the tumor vascular wall into the tumor tissue more easily [145]. Oncolytic reovirus pretreatment can improve the accumulation and distribution of PEG-liposomes in tumors. In mouse model experiments, researchers observed that when OVs were pre-treated with nanoparticles, the distribution and accumulation of liposomes in tumors increased 5.6-fold and 2.9-fold, respectively, enhancing the anti-tumor efficacy of the liposome-delivered drugs [146].

Other research has found that loading OVs into liposomes has significant anti-tumor effects. The genetically modified Newcastle disease virus exhibits selective toxicity in tumor cells and the liposome (vessel-targeting liposome) targets vascular endothelial cells, which can accurately deliver the Newcastle disease virus to the tumor site, significantly prolonging the survival time of mice [147]. This combination promotes interaction between the virus and immune cells, improves the activity of immune cells and anti-tumor immune responses, and has significant clinical value.

In response to many tumors’ low immunogenicity, which makes it difficult for the immune system to recognize and attack them, researchers have prepared CCL2-loaded liposomes to encapsulate the OAds into liposomes and then injected them into mice. Research has found that this new immunotherapy strategy can effectively enhance the infiltration and activation of monocytes in tumor tissues, as well as enhance the anti-tumor immune response, and inhibit tumor growth [67].

These experiments all have a certain degree of innovation and practicality, proving the feasibility of combining liposome nanomaterials with OVs. However, currently, it only stops at mouse experiments. Issues related to side effects and safety for clinical use remain to be investigated.

MSCs have emerged as promising delivery vehicles for OVs. While MSCs are macromolecular and not classified as nanomaterials, their distinctive cellular properties and secretion capabilities facilitate the transport of OVs directly to tumor sites, offering a novel avenue for cancer therapy [148,149,150]. Additionally, the surface adhesion proteins of MSCs can bind with nanoparticles, effectively cloaking the nanoparticles. This interaction enhances the stability of the delivery system and optimizes the release of therapeutic agents [151].

In addition, the Extracellular Vesicles (EVs) secreted by MSCs are small-molecule substances that can be utilized for the treatment of numerous diseases, such as tumors, heart disease, liver disease, and neurological diseases [152]. Ranging from 10 to 1000 nanometers, these vesicles are pivotal in cellular function regulation and signal transduction, mediating the transfer of bioactive molecules—proteins, nucleic acids, and metabolites—across cells. Their intrinsic targeting ability and membrane permeability allow EVs to deliver these molecules precisely to designated cells. For example, cancer cell-derived EVs can modulate the extracellular matrix via the Transforming Growth Factor-β signaling pathway, thus fostering the proliferation and invasion of tumor cells in nascent neoplastic tissues [153]. EVs could enclose bioactive molecules within their vesicles to protect them from degradation and clearance, which greatly contributes to their advantageous use in combination with OVs. Studies indicate that even when large biopharmaceutical OVs are loaded into EVs, their charge and size characteristics do not significantly change, and their antitumor activity is enhanced compared to viruses or EVs alone [154].

In one study, the authors explored the use of extracellular vesicles to encapsulate OVs and enhanced the targeted delivery and therapeutic effect of the virus through cytokine conditioning [155]. EVs may also be involved in the radiative long-distance effect of the OAds Telomelysin (OBP-301). EVs isolated from the supernatant of HCT116 human colon cancer cells treated with OBP-301 were confirmed to contain OBP-301. After observing that these EVs demonstrated similar cytotoxic activities (apoptosis and autophagy) as OBP-301, an intra-tumor injection was performed in a bilateral subcutaneous HCT116 and CT26 tumor model, revealing a potent antitumor effect of OB-301 on tumors not directly treated. This effect was found to be directly mediated by tumor-derived EVs containing OBP-301, and tumor-derived EVs displayed high tumor tropism in rectal in situ tumors of HCT116 [156].

The formation of EVs released from cancer cells infected with OAds (IEVs, infection-derived EVs) and the changes in viral cargo over the course of the infection were investigated. IEVs were secreted before the release of OV’s progeny and had structures similar to those of normally secreted EVs, suggesting they are more than just apoptotic fragments from infected cells. IEVs could carry the viral genome and induce infection in other cancer cells. Therefore, the role of EVs in the adenovirus life cycle may be a crucial component of successful infection and could be used in cancer therapy and gene therapy [157].

**Figure 4:**
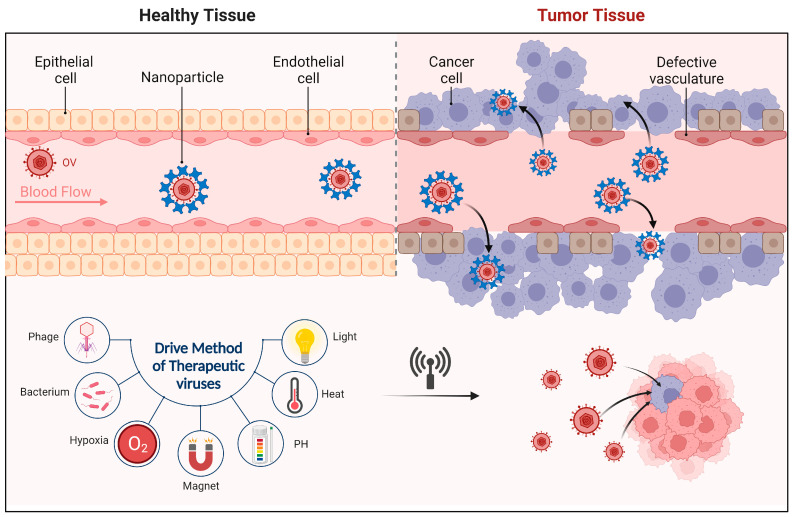
Many studies have employed various novel nanomaterials to ‘precisely guide’ viruses to tumor tissues using elements such as light, heat, magnetism, and pH. It’s akin to equipping the virus with a ‘signal localization’ function that allows it to accurately navigate to the designated location without impacting normal tissues.

Besides loading OVs into liposomes or exosomes, another approach is to pair OVs with novel nanoparticles to further enhance therapeutic outcomes, as shown in Figure 4.

In the aforementioned co-application of CIK cells and OVs, researchers used a biocompatible hydrogel as a carrier to simultaneously deliver CIK cells and OAds carrying IL12 and IL15 to the tumor site. Experimental results showed that this hydrogel could protect CIK cells and OAds from attacks by the immune system, while also increasing their persistence in the body. Additionally, the OAds carrying IL12 and IL15 can enhance the activity of CIK cells, thereby enhancing the therapeutic effect on tumors.

In another study, researchers used a natural polymer, hyaluronic acid-modified thiolated chitosan (HA-SH), as a nano-delivery system to specifically deliver the measles virus-based oncolytic virus (OMV) vaccine to prostate cancer cells. The research found that OMV vaccines encapsulated in HA-SH nanoparticles exhibited increased cytotoxicity and viral infectivity, and could enhance the extracellular release rate and specificity of the vaccine while reducing cytotoxicity. The researchers further demonstrated the specificity and safety of the OMV vaccine encapsulated in HA-SH nanoparticles and showed its anti-tumor effect in a mouse model [74].

Furthermore, through in vitro screening experiments based on virus-encoded artificial microRNAs, a unique artificial microRNA (amiRNA) was discovered that provides a replication advantage for the VSVΔ51 OV platform. Validation of amiR-4 targets revealed that the protein ARID1A, involved in chromatin remodeling, plays an essential role in OVs replication resistance. Virus-directed targeting of ARID1A combined with small-molecule inhibition of the methyltransferase EZH2 achieves synthetic lethality in both infected and uninfected tumor cells. Uninfected cell bystander killing was observed to be mediated by the intercellular transfer of extracellular vesicles carrying amiR-4 cargo. The study proved that OVs can serve as replicating carriers for amiRNA therapy and have the potential to be combined with small molecules and immune checkpoint inhibitor therapy [158].

In addition, a dual-sensitive STAT3 inhibitor nano-prodrug was developed. The inception of nano prodrugs aimed to elevate the precision of cancer therapy, all without causing harm to normal tissues. By harnessing the heightened presence of reactive oxygen species and glutathione within the tumor microenvironment, this concept spurred the development of a wide range of nanomedicines tailored for optimized drug release specifically at the tumor location [159]. When used in combination with OVs, it was able to induce pyroptosis in cancer cells and trigger immune responses, significantly enhancing anti-tumor effects. In this experiment, it was noted that when the oncolytic herpes simplex virus was combined with mesoporous silica nanoparticles, it triggered the release of substances carried within the mesoporous silica nanoparticles. These substances have the potential to interact with signaling pathways within cancer cells, resulting in the activation of Gasdermin D (GSDMD). Upon activation, GSDMD changes its molecular structure, equipping it with the capability to disrupt and rupture cell membranes.

This phenomenon may initiate a process known as pyroptosis, which is a form of programmed cell death characterized by cell membrane rupture, an inflammatory response, and the activation of GSDMD. Pyroptosis represents a specific type of regulated cell death that is induced by the activation of inflammasomes. The initiation of inflammasomes involves two key signals: an initial signal triggered by NF-κB that leads to the transcriptional upregulation of inflammasome components, and a sensing signal that subsequently triggers pyroptosis through the actions of pro-inflammatory cysteine proteases. GSDMD is cleaved by enzymes such as CASP11 or CASP1, resulting in the generation of two fragments—a 22 kDa C-terminal fragment (GSDMD-C) and a 31 kDa N-terminal fragment (GSDMD-N). GSDMD-N relocates to the inner leaflet of the plasma membrane, where it binds to phospholipids, disrupting the membrane’s normal permeability barrier and ultimately leading to membrane rupture [160,161].

The STAT3 inhibitor and molecular switch were combined, allowing the drug to undergo dual-responsive release within cancer cells. The researchers applied this nano-prodrug in combination with OVs in vitro and in vivo tumor models to observe its anti-tumor effects. The results indicated that the nano-prodrug significantly inhibited STAT3 activity, suppressing cancer cell growth and metastasis. Simultaneously, the nano-prodrug induced thermal coagulation, causing cancer cell death and releasing heat shock proteins, which in turn activated dendritic cells and T-cell immune responses. In the in vivo tumor model, this nano-prodrug combined with OVs significantly inhibited tumor growth and metastasis, while inducing thermal coagulation and immune responses in tumor cells, promoting T-cell infiltration and tumor cell apoptosis [162]. The experimental data confirmed that this combined therapy could significantly inhibit tumor growth and trigger strong immune responses, potentially providing a new direction for tumor treatment. It is believed that with more preclinical and clinical trials, even more potential will be unearthed.

Another strategy involves improving the immune-suppressive effects of OAds therapy by utilizing a bio-reducible polymer-mediated delivery method. This method uses a bio-reducible polymer to deliver OAds to tumor cells, reducing anti-viral immune responses while enhancing anti-tumor immune responses. In a mouse model, this method effectively inhibited tumor metastasis [163].

Further advancements in carrier strategies involve using genetic engineering to encapsulate OAds in cell membrane nanovesicles, thereby creating a novel tumor therapy platform. Experimental results showed that these genetically engineered cell membrane nanovesicles not only protect OAds from the immune system but also increase their stability and concentration in the body, effectively killing tumor cells [164].

Significant advances have been achieved with inorganic carriers as well. Researchers have developed calcium-phosphate biomineralized OVs by integrating phosphate-bearing OV with calcium compounds, enhancing the viruses’ stability and activity [165].

Furthermore, some researchers modified the calcium-phosphate biomineralized OVs by adding magnetic iron oxide nanoparticles and targeting peptides on the surface of human osteosarcoma cells, giving it dual-mode imaging and tumor cell-targeting functions [165]. In an innovative approach, viruses were directed to target cells using externally guided physical signals. Nanomagnets extracted from magnetic bacteria were used to help the virus better cross the vessel wall and enter tumor tissues. These nanomagnets can guide the virus accurately to the tumor location under the influence of an external magnetic field, thereby enhancing therapeutic effects [166]. Researchers also modified the M13 virus so it could be stimulated by light to release therapeutic agents, in conjunction with a remote-controlled optical system. This system will be controlled by a wireless remote control, allowing patients to receive painless treatment. The corresponding animal experiments demonstrated its potential in treating cancer cells [167].

### 5.2. Combination of Viral Vectors and Nanobiomaterial Delivery Systems

#### 5.2.1. Advantages of Combination

Viral vectors and nanomaterials have been engineered by scientists to act as precise drug-delivery tools [168,169]. They can specifically identify and bind to tumor cells, enhancing the efficacy of drugs through this precision targeting. This not only intensifies the therapeutic effect of the drugs but also significantly reduces their toxicity and side effects on healthy cells, thus improving the safety and comfort of treatment [170,171].

Additionally, nanomaterials play a crucial protective role during drug delivery [172]. They can prevent the drug from being degraded or cleared by enzymes within the body before it reaches its target. Furthermore, the unique properties of nanomaterials allow us to control the rate at which drugs are released, maintaining an effective concentration of the drug within the body to ensure the maximum therapeutic effect [173]. For some hard-to-reach tumors, such as those behind the blood-brain barrier, nanomaterials have demonstrated irreplaceable functions. They can assist drugs in penetrating these physiological barriers, enabling drugs to effectively target hard-to-reach tumor regions [174]

Simultaneously, viral vectors are designed to deliver genes that stimulate immunity [175,176]. These genes can encode tumor antigens, triggering or enhancing the immune system’s response, such as increasing T-cell activity, thereby improving therapeutic outcomes. For instance, adeno-associated viruses are regarded as one of the most promising vectors for gene therapy [177]. The modern medical field is gradually transitioning towards more personalized and precision-based treatment models. In this process, nanomaterials and viral vectors are utilized in disease monitoring and diagnosis. For instance, fluorescent nanoparticles can be used to track drug distribution and release, aiding in assessing therapeutic effectiveness [178].

The applications of viral vectors and nanomaterials are not limited to single treatment modes. They could be used in combination therapies, such as simultaneously delivering chemotherapy drugs and gene therapy [179]. For example, adeno-associated viruses enveloped within extracellular vesicles can facilitate improved delivery of therapeutic genes to the heart [180]. This synergistic effect can enhance therapeutic results, reduce side effects, prevent the development of drug tolerance, and provide new possibilities for cancer treatment.

#### 5.2.2. Current Research on Combined Applications

Numerous studies have confirmed that the combination of nanoparticles with viral vectors can further enhance targeting specificity and drug stability.

Early research developed a self-assembling nanoscale adenovirus with a synthetic lipid envelope to enhance penetration into tumor spheroids and similar entities [181]. Subsequently, many novel combinations of nanomaterials with viral vectors have emerged. For instance, the formation of peptide/DNA fibers from self-assembling amphiphilic peptide units and supercoiled circular plasmid DNA has included viruses, successfully preserving the biological activity of the virus [182]. Mesoporous silica nanoparticles (MSNP) loaded with cargo, functionalized with a biological coating, exhibit molecular assembly. The MSNP can serve as a delivery system, given its porous structure that allows for a high therapeutic payload, while TMV can act as a biocompatible coating to enhance cell interactions [183]. However, issues have arisen, such as the internal payload capacity of Physalis mottle virus (PhMV) like nanoparticles being limited due to the presence of a single reactive cysteine (C75) in each capsid protein, coupled with inherently low reactivity [184].

In recent years, the combined use of nanoparticles and viral vectors has played an even more significant role. Optimized DOTAP: DMPC lipid emulsions, due to their low cytotoxicity and unique ability to encapsulate viral particles, have been employed for adenovirus gene delivery, highlighting the importance of nanoparticle formulations [185]. Kernan and colleagues developed a nanoparticle drug delivery system utilizing the nucleoprotein component of TMV. These high aspect ratio soft nanorods formed by TMV, due to their simple genetic and chemical engineering, tunable size and shape, and biocompatibility, have to some extent overcome the off-target effects and chemotherapy-related complications that remain significant challenges in the treatment of non-Hodgkin’s lymphoma [186].

Moreover, research on adeno-associated virus vectors has led scientists to develop a polymeric micelle system. Its significant penetrative effect can activate a suicide gene in mouse models with pancreatic cancer, achieving an anti-tumor effect [187]. Some scientists have invented the Ad@AuNPs complex, which facilitates cell adhesion and uptake, independent of Coxsackievirus and adenovirus receptors as well as integrins αvβ3 and αvβ5, significantly improving transduction without limiting the biological activity of Ad [188]. Other studies have explored the potential of using virus-like particles from the Flock House Virus (a member of the Nodaviridae family) combined with tumor-homing peptides as carriers for hydrophobic drug delivery [189]. This approach helps to address issues related to the encapsulation, specific delivery, safety, and immunogenicity of hydrophobic drugs. Utilizing this novel carrier, targeted therapy for glioblastoma, lung adenocarcinoma, and triple-negative breast cancer has shown promising results [89]. Additionally, Malogolovkin et al. have considered using viral vectors and nanoparticles for the delivery of optogenetic payloads and the activation of tumor fluorescence [178].

In summary, significant progress has been made by researchers in exploring the use of novel nanomaterials to coat viral vectors to enhance their penetrative properties, or to create nanoscale composite carriers to enhance targeted action. When comparing the therapeutic effects of nanomaterials with non-nanomaterials, nano therapy is generally considered more effective.

### 5.3. Combination of Vaccine Viruses and Nanobiomaterial Delivery Systems

Although various types of viruses can be used to make vaccines to prevent related cancers, they all have certain limitations. The production of VLPs is limited by production efficiency and scalability, and there are significant restrictions regarding VLPs production [190].

The use of nanomaterials greatly aids vaccine usage by increasing anti-tumor efficacy and durability. For instance, a nanoparticle-mediated delivery system has been developed for an orally administered vaccine induced by HPV [191]. For instance, in breast cancer vaccine research, researchers have combined nanomaterials with specific antigens to induce the production of an immune response, prolong the presence of antigens, and increase their targeting [192].

The use of nanotechnology means that we construct VLPs ourselves, and precise VLP architecture is very important for antigens to provoke protective antibody responses. Meanwhile, the creation of mosaic nanoparticles allows multiple antigens to be produced in a single particle, which is extremely significant for vaccine research [193]. VLPs, modified by various molecular techniques, are currently utilized as delivery vectors or platforms for the presentation of heterologous antigens, such as those derived from influenza. [194].Another study demonstrated the superiority of nanoparticles in treating cancer vaccines by applying VLPs to immunocompromised mice, resulting in effective systemic immunity [195].

Lundstrom and colleagues have found that alphavirus vectors can be utilized as recombinant viral particles or OVs, with encapsulation in nanoparticles allowing for the harnessing of RNA and DNA replicons, facilitating efficient gene delivery and tumor regression [196]. For many years, there has been an exploration into plants as a cost-effective and versatile platform for the production of vaccines and other biopharmaceuticals. Plant viruses have also been engineered to express subunit vaccines or serve as epitope presentation systems. Research has been extended to icosahedral and helical rod-shaped plant viruses [197]. Concurrently, virus-derived self-assembling protein nanoparticles (NPs) have emerged as an attractive antigen delivery platform for the development of both prophylactic and therapeutic vaccines. In the study by Zheng et al., the norovirus S domain (Nov-S) genetically engineered with a C-terminal SpyCatcher003 fusion has been developed into a robust, modular, and multifunctional NP-based carrier platform (Nov-S-Catcher003), enabling NPs to be readily equipped with SpyTag003-related antigens in a plug-and-play manner [198].

The combination of nanomaterials and immunoviruses holds tremendous potential for future cancer vaccine development.

### 5.4. Challenges and Limitations of Combination Therapy

The combination of OVs with nanomaterials undoubtedly represents a novel potential approach for tumor eradication. However, due to the heterogeneity and complexity of cancerous tissues, as well as the difficulty in controlling drug carriers and their specific functions, the synergistic use of nanomaterials with OVs still encompasses unknowns [199,200]. In tumor therapy, the consideration extends beyond merely removing cancerous tissues to the timely treatment and even prevention of the genesis of malignant cells. Furthermore, variations in nanomaterials, such as particle size, may also contribute to increased uncertainty in their biological distribution. Moreover, the differing properties between many viruses and nanomaterials, particularly in terms of metabolic stability, delivery stability, and hydrophobicity, are not fully understood regarding their mutual influence. Determining how to make combination therapies more effective and safer for patients is a primary concern that must be addressed by every researcher. Additionally, although the potential benefits of these emerging technologies are substantial and could represent a breakthrough in cancer treatment, during development, it is imperative to balance potential risks and ethical considerations to ensure their safe and equitable use [201].

## 6. Conclusions

In tumor treatment research, the combination of therapeutic viruses and nanomaterials has shown significant efficacy, marrying the targeting capabilities of viruses with the superior drug delivery of nanomaterials for a potent anti-cancer strategy. This innovative approach heralds a shift in cancer treatment paradigms.

Meanwhile, challenges persist. Enhanced precision in tumor targeting is required, demanding sophisticated developments in virus and nanomaterial design to reduce collateral damage to healthy cells. Additionally, increasing the drug-carrying capacity of these agents could improve therapeutic outcomes without increasing dosages. Addressing immune responses to these treatments is also paramount.

Future research is poised to tackle these hurdles, with the potential to make virus-nanomaterial combinations a cornerstone in cancer therapy. This interdisciplinary field spans biology, medicine, materials science, and nanotechnology, and it thrives on collaborative, cross-disciplinary efforts. Through continued innovation, we aim to deliver new treatment avenues for cancer patients and broader insights into disease management.

## Figures and Tables

**Table 1:** Overview of Therapeutic Viruses: Structure, Characteristics, Advantages, Related Treatments and Tumor Associations.

Virus	Structure	Characteristic	Advantage	Related Treatments	Tumor Associations	Refs.
Adeno-Associated Virus	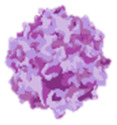	The helper virus replicates, infects, and lyses host cells only in the presence of the helper virus.	It has the advantages of strong safety, simple structure, and wide host, and as a prokaryotic virus, it is easier to eliminate the natural tropism of mammalian cells and bind to its receptors.	Luxturna, Zolgensma	Bladder cancer, prostate cancer, Kaposi’s sarcoma	[28,29]
Lymphocytic Choriomeningitis Virus	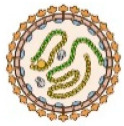	It replicates, and transcription and replication are mainly dependent on the polymerase synthesized by the virus itself.	The simplicity and rapid ability of vector generation to stabilize attenuation could elicit CTL responses of high intensity and cytolytic capacity.	Junin arenavirus live attenuated vaccine Candidate #1	Lung cancer, Colorectal cancer, Breast cancer, Pancreatic cancer	[30]
Epstein-Barr Virus	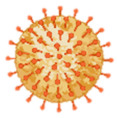	The ability to cause sarcoma necrosis, regression of transplantable tumors, and death of several tumor cell lines.	Leaving most normal cells unaffected and with little species specificity. Efficient transfection of relevant clay particles.	Gene modification	Nasopharyngeal carcinoma, Lymphoma	[31]
Adenovirus Vector ZD55	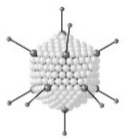	ZD55 has one cloning site to insert recombinant genes, ZD55 has elements of only Adv5.	The weak toxic effect of the virus was enhanced by the expression of the antitumor transgene.	ZD55-IL-24	Hepatoma, Breast cancer	[32]
M13 Bacteriophage	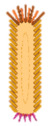	It infects only bacterial cells and lacks native tropism to normal tissues in humans and eukaryotes in general.	It is safe and has good tumor selectivity.	Gene modification	Soft tissue sar coma, Melanoma, Breast cancer, prostate cancer, pancreatic cancer	[33]
Phage	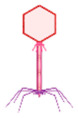	Phages pervade the human body, transcytosing human tissues efficiently and crossing the blood-brain barrier.	Safe and non-pathogenic nature. Most phages range in the nanoscale diameter. Phages in general lack diversity in their surface architecture. Phages are known to modulate innate as well as humoral immunity.	SP94-targeted virus, T-VEC	Breast cancer, prostate cancer, Chondrosarcoma	[34]

**Table 2:** Advantages of biological nanocarrier materials in the field of drug delivery and gene therapy and related treatments.

Name	Diagram	Features	Advantage	Related Treatments	Tumor	Ref.
Immune stimulating Nano adjuvants	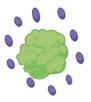	Influence immune response through the effect of antigen availability over time	Enhance antigenicity with weak antigenic substances, increase the level of specific circulating antibodies or produce effective protective immunity, change the produced, enhance cell-mediated hypersensitivity response, protect antigens from the decomposition of enzymes in the body	Nasal vaccines	Enhance the efficacy of cancer immunotherapy	[101]
Liposomal vaccine	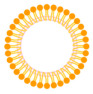	Developed to target specific immune cell types to induce certain immune responses	Ability to encapsulate and deliver vaccines to specific locations in the body and release their contents at specific	For the treatment of important viral, bacterial, fungal, and parasitic infections (including tuberculosis, tuberculosis)	A promising mRNA vaccine delivery vector that can effectively elicit tumor-specific cytotoxic T lymphocyte responses	[102]
Polymer vaccine	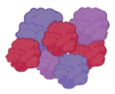	Protect antigens from degradation and prolong the residence time of antigens at the target site	It can prove the safety of vaccines and can be used to deliver immune adjuvants and achieve sustained release of vaccine	As a therapeutic agent delivery system for oncology treatment	The most attractive candidate for polymer nanoparticles in tumor immunotherapy	[103]
Virus-like particles	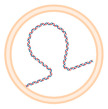	A virus-derived structure consisting of one or more different molecules with self-assembly ability, with the ability to self-assemble, mimicking the form and size of viral particles, but lacking genetic material	Storing immunogenicity and biological activity	CRISPR/Cas9 mRNA can be delivered for safe and efficient in vivo gene editing	It plays an important role in the prevention and treatment of infectious diseases and cancer	[94]
Porous silicon particles	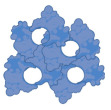	Sustained release of antiretroviral drugs	It can be used as a vehicle for the continuous release and processing of tumor antigens	Incorporated into tablets, thereby providing a sustained release of the drug	Prevention of HIV	[104]
Selenium nanoparticles	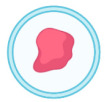	Has significantly reduced toxicity	Nanoselenium has better antioxidant capacity than other chemical forms of selenium and has important antibacterial activity against pathogenic bacteria, fungi, and parasites	It is used for various oxidative stress and inflammation-mediated diseases such as arthritis, cancer, diabetes, and kidney disease	Achieve precise anti-cancer treatment	[105]

## Data Availability

Data is contained within the article.
